# Supplementary material for: Participatory action research to address lack of safe water, a community-nominated health priority in rural South Africa
Source: PLoS One. 2023 Jul 27;18(7):e0288524. doi: 10.1371/journal.pone.0288524 (PMC10374036; doi:10.1371/journal.pone.0288524)
Supplement: Attachment — Submitted filename: Response to Review_03 May 23.docx [file pone.0288524.s001.docx]

| Reviewer(s)' Comments to Author(s): | Author response | Page |
| --- | --- | --- |
| Reviewer #1: |  |  |
| This paper provides as a case study from South Africa on community participatory research, and feedback on what interventions were ultimately possible and which did not succeed. The paper also provides a strong case for better listening to communities on what their problems are, and also realistic feedback that not all of these problems may be able to be solved as an outcome | Many thanks for your careful review and insightful comments. We have carefully addressed all the comments and suggestions and we believe that the revised version is now suitable for publication in PLOS ONE. We provide responses below |  |
| 1. Additional background information is needed on why a lack of access to safe water remains in the study area. Is it due to rolling blackout of power which impacts the water pumping, or is there simply not enough water quantity in the area available? South African citizens are guaranteed water so how does this fit into the PAC? | The resubmitted manuscript now includes additional background information on the persistent challenge of water access in the Bushbuckridge local municipality study area. While recent power cuts may be a contributing factor, they are not the primary cause of the water access issue in the region. Data collection for cycle one was conducted from 2017 -19, during which the situation was challenging, but it was no acute as it is in 2023. | 5 |
| 2. Line 135 is unclear, why only one cycle and what are the other cycles? | In this paper we report on the first cycle which comprised three steps described below. These steps were crucial in informing the subsequent steps reported elsewhere. | 8 |
| 3. Why did the community want a recycling centre and beyond the presence of the NGO was there a community advocate? | These action items were developed in collaboration with local communities, NGOs, and researchers. Specifically, the NGO led the implementation of this particular action item in partnership with local communities. | 17 |
| 4. Line 403 is missing a reflection on the election cycle impacting the mayor. Was there any one community issue that the mayor supported more or less? | During the planning phase, it is crucial to time the project in a way that ensures the mayor's availability for support, buy-in, and participation, without other commitments. This is particularly important during elections when the mayor's availability may be limited. | 23 |
| 5. The paper is missing a discussion on the strengths of this stakeholder engagement timeline, because other work shows that when it is too fast it fails. An additional figure with a timeline would assist the reader in understanding the many listening sessions and final outcomes. | Agreed, and we have added more discussion on the strengths of stakeholder engagement timeline in the discussion section and a figure. However, this paper is reporting on the 1^st^ of 5 cycles of the wider programme of research. | 28 |
| 6. Was there any reflection on the international authorship team here and the community listening sessions? Or, that partnership model? | Thank you for these constructive suggestions. We have tried to address this by adding a researcher reflection in the result section. | 28 |
| 7. In figure 1 and 2 please write out acronyms in full, it is hard to follow them | Thank you, this has been corrected as suggested |  |
| 8. The results and discussion are well written but could be strengthen with more reflection on the “failures.” Can the authors give more details on outcomes not achieved and key points that can be pointed to when outcomes were achieved. Case studies such as this benefit literature the most in not overlooking both. | The authors thank the reviewer for urging us to incorporate these contextual features. The result and discussion section of the revised manuscript gives more details on the outcomes not achieved. |  |
| Reviewer #3 |  |  |
| I believe the breadth of this study is significant, and I highly suggest that the manuscript be published after minor adjustments. Thank you kindly. | Thank you for the encouraging comments and positive feedback, which has helped us to improve the quality of our manuscript. |  |
| 1. The manuscript should be checked carefully for English and formatting style | We have formatted and reviewed the manuscript. |  |
| 2. The authors should provide Figure no. 3? | Thank you. Figure 3 provided |  |
| 3. The authors should reorganize the methods in simple way? | We have reviewed the methods section and added stakeholder timeline diagram. |  |
| 4. The authors should provide flowchart summarize the study design? | Thank you for this suggestion, we have added a schematic illustration of the methods. |  |
|  |  |  |

END OF RESPONSE TO REVIEW
